# *Flavobacterium plurextorum* sp. nov. Isolated from Farmed Rainbow Trout (*Oncorhynchus mykiss*)

**DOI:** 10.1371/journal.pone.0067741

**Published:** 2013-06-25

**Authors:** Leydis Zamora, José F. Fernández-Garayzábal, Cristina Sánchez-Porro, Mari Angel Palacios, Edward R. B. Moore, Lucas Domínguez, Antonio Ventosa, Ana I. Vela

**Affiliations:** 1 Centro de Vigilancia Sanitaria Veterinaria (VISAVET), Universidad Complutense, Madrid, Spain; 2 Departamento de Sanidad Animal, Facultad de Veterinaria, Universidad Complutense, Madrid, Spain; 3 Piszolla, S.L., Alba de Tormes, Salamanca, Spain; 4 Departamento de Microbiología y Parasitología, Facultad de Farmacia, Universidad de Sevilla, Sevilla, Spain; 5 Culture Collection University of Gothenburg (CCUG) and Department of Infectious Disease, Sahlgrenska Academy of the University of Gothenburg, Göteborg, Sweden; Beijing Institute of Microbiology and Epidemiology, China

## Abstract

Five strains (1126-1H-08^T^, 51B-09, 986-08, 1084B-08 and 424-08) were isolated from diseased rainbow trout. Cells were Gram-negative rods, 0.7 µm wide and 3 µm long, non-endospore-forming, catalase and oxidase positive. Colonies were circular, yellow-pigmented, smooth and entire on TGE agar after 72 hours incubation at 25°C. They grew in a temperature range between 15°C to 30°C, but they did not grow at 37°Cor 42°C. Based on 16S rRNA gene sequence analysis, the isolates belonged to the genus *Flavobacterium*. Strain 1126-1H-08^T^ exhibited the highest levels of similarity with *Flavobacterium oncorhynchi* CECT 7678^T^ and *Flavobacterium pectinovorum* DSM 6368^T^ (98.5% and 97.9% sequence similarity, respectively). DNA–DNA hybridization values were 87 to 99% among the five isolates and ranged from 21 to 48% between strain 1126-1H-08^T^, selected as a representative isolate, and the type strains of *Flavobacterium oncorhynchi* CECT 7678^T^ and other phylogenetic related *Flavobacterium* species. The DNA G+C content of strain 1126-1H-08^T^ was 33.2 mol%. The predominant respiratory quinone was MK-6 and the major fatty acids were iso-C_15∶0_ and C_15∶0_. These data were similar to those reported for *Flavobacterium* species. Several physiological and biochemical tests differentiated the novel bacterial strains from related *Flavobacterium* species. Phylogenetic, genetic and phenotypic data indicate that these strains represent a new species of the genus *Flavobacterium,* for which the name *Flavobacterium plurextorum* sp. nov. was proposed. The type strain is 1126-1H-08^T^ ( = CECT 7844^T^ = CCUG 60112^T^).

## Introduction

The genus *Flavobacterium* is the type genus of the family *Flavobacteriaceae* accommodating Gram-negative, non-endospore-forming, aerobic, oxidase-positive, non-fermenting, predominantly gliding, yellow-pigmented bacteria [1], [2]. The genus, initially described to accommodate seven species, has considerably expanded with the description of many new species. Currently it includes 99 species, many of them described during the last five years. [3]. Members of the genus *Flavobacterium* can been isolated from a number of diverse habitats such as soil, water, sludge, plants, food products such as fish, meat, poultry, milk or lactic acid beverages [2], [4]. Most species are non-pathogenic, although a number of species have been associated with different clinical infections, being freshwater fish the animals most prone to flavobacterial infections [5]. Some *Flavobacterium* species, mainly *Flavobacterium columnare*, *Flavobacterium branchiophilum* and *Flavobacterium psychrophilum,* are well-recognized fish pathogens responsible for important economic losses in the fish farming industry [6], [7]. However, several other species such as *Flavobacterium hydatis*, *Flavobacterium jhonsoniae*, *Flavobacterium succinicans, Flavobacterium chilense*, *Flavobacterium araucananum* or *Flavobacterium oncorhynchi* have been also associated with infections in fish [1], [4], [5], [8]–[10]. Additionally, a number of new *Flavobacterium* species also have been described from the water of aquaculture facilities [11]–[13]. This plethora of *Flavobacterium* species could reproduce the diversity of flavobacteria associated with fish or fish surrounding environments. Some of these species could be considered commensal and opportunistic pathogenic bacteria [4], which point out the necessity for an accurate identifications of those strains of *Flavobacterium* spp. isolated from fish or fish farm environments. However, such identifications are extremely difficult based exclusively on biochemical criteria [4], [8], [14] and must be complemented with chemotaxomic and genetic methods [4], [5].

In this article, we report the phenotypic, genotypic and phylogenetic characterization of five novel *Flavobacterium-*like strains isolated from diseased trout. Based on the presented findings, a new species of the genus *Flavobacterium*, *Flavobacterium plurextorum* sp. nov., is proposed.

## Materials and Methods

The present work does not include any experimental infections trial with farmed trout, just trout exclusively were used to identify microbiologically the etiological agent of the bacterial septicemia. Therefore, we did not consult with the IACUC and no specific national regulations for these procedures are available. Nevertheless, in order to ensure the welfare and ameliorate suffering of trout during transportation to the laboratory and euthanasia, trout were handled according to guidelines of relevant international organisms such as OIE (http://www.oie.int/doc/ged/D7821.PDF) and AVMA (https://www.avma.org/KB/Policies/Documents/euthanasia.pdf) and they were further necropsied under aseptic conditions. In addition, these procedures were approved by the responsible of animal welfare of the UCM Animal Health Department. The trout were sacrificed for the purpose of the study and the sacrifice was approved by the Technical Manager (Mari Angel Palacios, DVM, PhD) of the fish farm located in the west of Spain.

### Trout and Strain Isolation

A clinical episode of septicemia occurred in a rainbow trout (*Oncorhynchus mykiss*) farm located in the central region of Spain. Affected trout were submitted by the Technical Manager of the fish farm to the Animal Health Surveillance Centre (VISAVET) of the Universidad Complutense (Madrid, Spain) for a confirmatory microbiological diagnosis.

Five Gram-negative, rod-shaped bacteria were isolated from liver (strains 986-08 and 424-08), gills (strains 1084B-08 and 51B-09) and eggs (1126-1H-08^T^) of five different trout. The strains were recovered in two different years (2008 and 2009) and they were isolated on tryptone glucose extract agar (TGE; Difco) after incubation at 25°C for 72 hours under aerobic conditions.

### Phylogenetic Analysis

A large continuous sequence (approximately 1,400 bases) of the 16S rRNA gene of five strains was determined bidirectionally using universal primers pA (5′- AGAGTTTGATCCTGGCTCAG, positions 8–27, *Escherichia coli* numbering) and pH* (5′-AAGGAGGTGATCCAGCCGCA, positions 1541–1522, *E. coli* numbering) as described previously [10], and subjected to a comparative analysis. The identification of the phylogenetic relatives and calculations of pair-wise 16S rRNA gene sequence similarities were achieved, using the EzTaxon-e server [15]. The 16S rRNA gene sequences of the type strains of all validly published species of the genus *Flavobacterium* were retrieved from GenBank and aligned with the newly determined sequences using the program SeqTools [16]. Phylogenetic trees were constructed according to three different algorithms: neighbour-joining [17], using the programs SeqTools and TREEVIEW [18]; maximum-likelihood, using the PHYML software [19]; and maximum-parsimony, using the software package MEGA (Molecular Evolutionary Genetics Analysis) version 5.0 [20]. Genetic distances for the neighbour-joining and the maximum-likelihood algorithms were calculated by the Kimura two-parameter [21] and close-neighbour-interchange (search level = 2, random additions = 100) was applied in the maximum-parsimony analysis. The stability of the groupings was estimated by bootstrap analysis (1000 replications).

### Genomic DNA G+C Content Determination and DNA-DNA Hybridizations

The G+C content of the genomic DNA of a representative strain (1126-1H-08^T^) was determined from the mid-point value (Tm) of the thermal denaturation profile [22], obtained with a Perkin-Elmer UV-Vis Lambda 20 spectrophotometer at 260 nm.

Genomic DNA-DNA hybridizations were carried out between strains 1126-1H-08^T^, 986-08, 424-08, 1084B-08 and 51B-09, and between strain 1126-1H-08^T^ and the type strains of the closest phylogenetically related species. DNA was extracted and purified by the method of Marmur [22]. Hybridization studies were carried out, using the membrane method of Johnson [23], described in detail by Arahal *et al.* [24]. The hybridization experiments were carried out under optimal conditions, at a temperature of 44°C, which is within the limits of validity for the membrane method [25]. The percentages of hybridization were calculated as described by Johnson [26]. Three independent determinations were carried out for each experiment and the results reported as mean values. The type strains of species *F. aquidurense* CCUG 59847^T^, *F. araucananum* CCUG 61031^T^, *F. hydatis* DSM 2063^T^, *F. pectinovorum* CCUG 58916^T^, *F. frigidimaris* CCUG 59364^T^, *F. chungangense* CCUG 58910T and *F. oncorhynchi* CECT 7678^T^ were included in this study.

### Chemotaxonomic Characteristics

Respiratory quinones of strain 1126-1H-08^T^ were extracted from 100 mg of freeze-dried cell material, using the two stage method described by Tindall [27], [28], and further separated by thin layer chromatography on silica gel and analyzed, using HPLC, by the identification service of the DSMZ (Braunschweig, Germany).

For cell fatty acid-fatty acid methyl ester (CFA-FAME) analyses, strain 1126-1H-08^T^ was grown on Columbia II agar base (BBL 4397596) with 5% horse blood, at 30°C for 30–48 h, under aerobic conditions. The CFA-FAME profile was determined using gas chromatography (Hewlett Packard HP 5890) and a standardized protocol similar to that of the MIDI Sherlock MIS system [29], described previously [10]. CFAs were identified and the relative amounts were expressed as percentages of the total fatty acids of the respective strains.

### Morphological, Physiological and Biochemical Characteristics

The minimal standards for the description of new taxa in the family *Flavobacteriaceae* [30] were followed for the phenotypic characterization of the strains. Gram-staining was performed as described by Smibert & Krieg [31]. Oxidase activity was determined by monitoring the oxidation of tetramethyl-*p*-phenylenediamine on filter paper and catalase activity was determined, using 3% H_2_O_2_ solution [31]. Hydrolysis of L-tyrosine (0.5%, w/v), lecithin (5%, w/v) [31], esculin (0.01% esculin and 0.05% ferric citrate, w/v), gelatin (4%; w/v), starch (0.2%, w/v), and casein [50% skimmed milk (Difco), v/v] were tested using nutrient agar as basal medium [30]. DNase test agar (Difco) was used for the DNase assay. Hydrolysis of urea (1%, w/v) was tested as described by Bowman *et al.* [32]. Growth in brain heart infusion broth was assessed at 15, 25, 30, 37 and 42°C, with 3.0, 4.5 and 6.5% added NaCl, and under anaerobic (with 4–10% CO_2_) and micro-aerobic (with 5–15% O_2_ and 5–12% CO_2_) conditions, using GasPak Plus and CampyPak Plus systems (BBL), respectively. Growth was tested on MacConkey (bioMérieux), nutrient (Difco) and trypticase-soy (bioMérieux) agar plates. The presence of gliding motility, using the hanging drop technique, and the production of flexirubin-type pigments and extracellular glycans were assessed, using the KOH and Congo red tests, respectively [1]. The strains were further biochemically characterized using the API 20NE and API ZYM systems (bioMérieux) according to the manufacturer’s instructions, except that incubation temperature was 25°C. The type strains of species *F. aquidurense* CCUG 59847^T^, *F. araucananum* CCUG 61031^T^, *F. hydatis* DSM 2063^T^, *F. pectinovorum* CCUG 58916^T^, *F. frigidimaris* CCUG 59364^T^, *F. chungangense* CCUG 58910^T^ and *F. oncorhynchi* CECT 7678^T^ were included in this study as references for the investigation of the phenotypic properties of the trout strains, using the same laboratory conditions.

### PGFE Typing

The five strains were characterized by pulsed-field gel electrophoresis (PFGE), after digestion of their genomic DNAs with the restriction enzymes *Bsp120*I and *Xho*I, according to the specifications of Chen *et al.* [33]. DNA fragments were resolved in a 1% agarose gel with a pulse-field gel electrophoresis apparatus, CHEF-DR III (Bio-Rad), at 6V/cm for 40 hours, with switching times ramped from 0.1 to 12 s at 14°C, with an angle of 120°. The gels were stained for 30 min with Syber-Safe and photographed under UV light (Gel-Doc, Bio-Rad). Strains differing in at least one band were considered different.

## Results and Discussion

**Figure 1:**
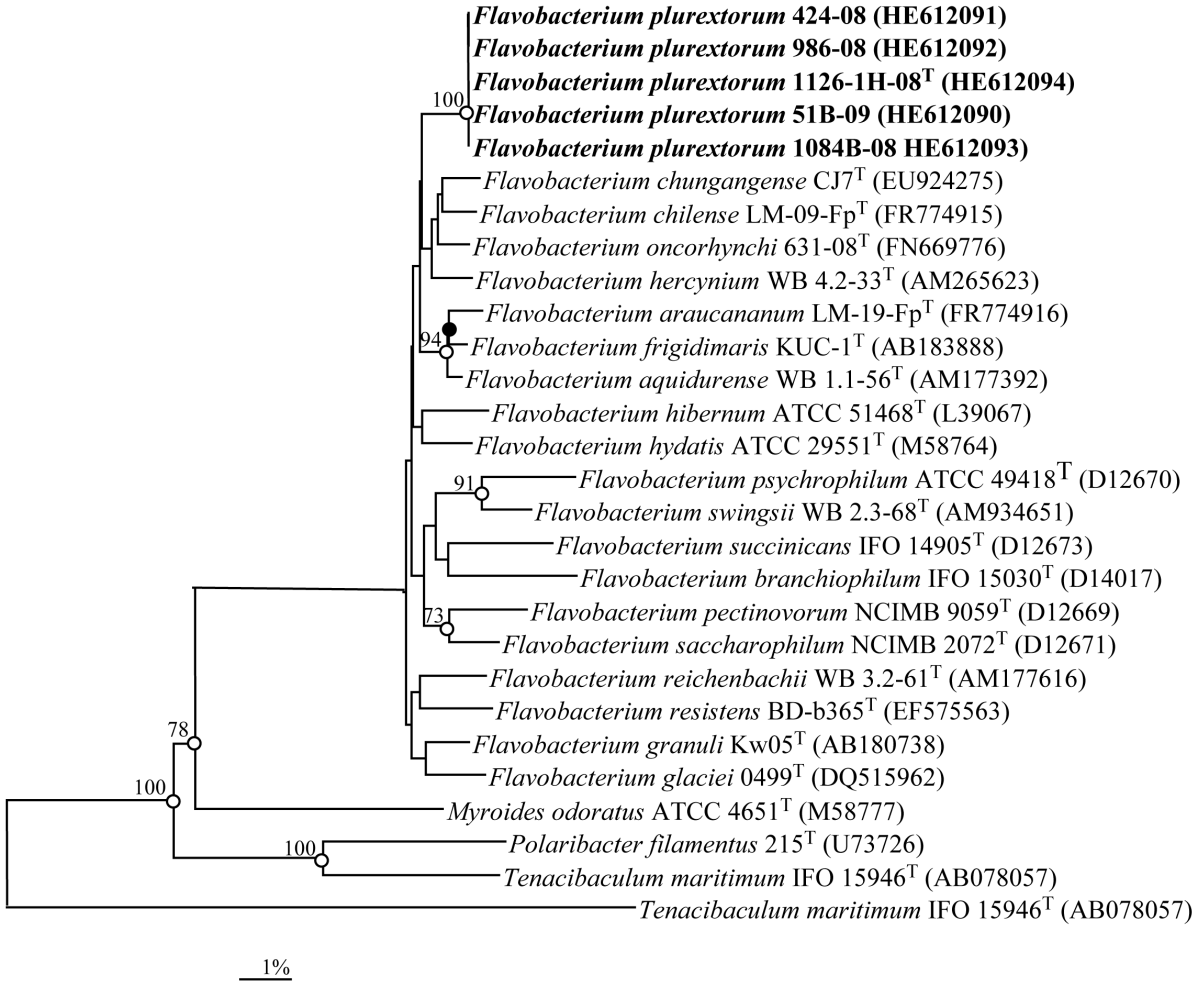
Phylogenetic tree based on 16S rRNA gene sequence comparisons, obtained with the neighbour-joining algorithm, showing the relationships of *Flavobacterium plurextorum* sp. nov. with related species. *Flexibacter flexilis* ATCC 23079^T^ was used as an outgroup. Bootstrap values (expressed as a percentage of 1,000 replications) greater than 70% are given at the nodes. Solid circles indicate that the corresponding nodes (groupings) are also obtained on the maximum-likelihood tree. Open circles indicate that the corresponding nodes (groupings) are also obtained on the maximum-likelihood and parsimony trees. Sequence accession numbers are indicated in brackets. Bar, 1% sequence divergence.

16S rRNA gene sequences were determined for the five trout strains, displaying 100% 16S rRNA sequence similarity among them. Sequence searches showed that the 16S rRNA gene sequence of the strains were most similar to those of species of the genus *Flavobacterium*, exhibiting the highest levels of similarity with the sequence of the type strains of *Flavobacterium oncorhynchi* CECT 7678^T^ and *Flavobacterium pectinovorum* DSM 6368^T^ (98.5% and 97.9% sequence similarity, respectively). In addition, strains exhibited 16S rRNA gene sequence similarities greater than 97.0% with other seventeen other *Flavobacterium* species. It is clear from the phylogenetic analysis (Fig. 1) that the trout strains held a clear affiliation to the genus *Flavobacterium* and represented a distinct sub-lineage clustering with a cluster of four species that included *F. pectinovorum, F. chilense, F. oncorhynchi* and *F. hercynium*. However, their position within this sub-group was not supported by significant bootstrap values. The GenBank accession numbers for the 16S rRNA gene sequences of five strains sequenced in this study are shown in Fig. 1.

Genomic DNA–DNA hybridizations between the trout strains yielded binding values of 87 to 100%. *Flavobacterium* species with 16S rRNA gene sequence similarities to the sequences of the trout strains lower than 98.0% correlated with levels of genomic DNA-DNA relatedness always lower than 70% [9]–[11], [34]–[36]. For that reason, DNA-DNA hybridizations were carried out only between strain 1126-1H-08^T^ and the type strains of the phylogenetically closest related species; *i.e.*, those species with 16S rRNA gene sequence similarities greater than 97.5%. The levels of DNA-DNA relatedness for strain 1126-1H-08^T^ with respect to *F. aquidurense* CCUG 59847^T^, *F. araucananum* CCUG 61031^T^, *F. hydatis* DSM 2063^T^, *F. pectinovorum* CCUG 58916^T^, *F. frigidimaris* CCUG 59364^T^, *F. chungangense* CCUG 58910^T^ and *F. oncorhynchi* CECT 7678^T^ ranged between 21 and 48%. These values were below the 70% cut-off point for species delineation [37], [38] and clearly confirmed that the trout strains belong to a distinct genomic species of the genus *Flavobacterium*. The DNA G+C content of strain 1126-1H-08^T^ was 33.2 mol%, a value consistent with those of the genus *Flavobacterium* [1], [30].

Chemotaxonomic characteristics of strain 1126-1H-08^T^ were in accordance with those of members of the genus *Flavobacterium* [5], [6]: the major quinone was MK-6 (95%) with minor amounts of MK-5 (5%). The predominant cell fatty acids of strain 1126-1H-08^T^ were iso-C_15∶0_ (19%) and C_15∶0_ (15%). Strain 1126-1H-08^T^ also contained moderate or small amounts of C_16∶1_ *ω7c* (10%), C_15∶1_ *ω6c* (9%), iso-C_15∶0_ 3-OH, C_17∶1_ *ω6c*, isoG-C_15∶1_ (6%/each), iso-C_17∶0_ 3-OH (5%), iso-C_17∶1_ *ω9c,* C_15∶0_ 3-OH, C_16∶0_ 3-OH (3%/each), isoaldehyde-C_15∶0_, C_16∶0_, iso-C_16∶0_ 3-OH, unknown fatty acids with an equivalent chain length of 11.5 (2%/each) and C_17∶1_ *ω8c*, iso-C16∶0, C_12∶1_, aldehyde-C_14∶0_, anteiso-C_15∶0_ and unknown fatty acids with an equivalent chain lengths of 14.8 and 12.5 (1%/each) (Table 1).

**Table 1:** Cellular fatty acid compositions of *Flavobacterium plurextorum* 1126-1H-08^T^ and its closest phylogenetic neighbours.

Fatty acid	1	2	3	4	5	6	7	8
**Saturated**								
C_12∶1_	1	tr	–	–	–	–	tr	–
C_14∶0_	tr	–	tr	1.1	tr	tr	–	tr
C_15∶0_	15	13.5	11.9	5.5	5.6	6.9	20.6	15.7
C_16∶0_	2	1.6	1.1	2.3	2.8	2.2	tr	2.9
**Hydroxy**								
C_15∶0_ 2OH	–	–	1.1	–	tr	–	–	–
C_15∶0_ 3OH	3	3.3	1.9	–	–	–	1.8	–
iso-C_15∶0_ 3OH	6	7.8	6.9	7.7	8.6	5.8	7.1	5.8
C_16∶0_ 3OH	3	–	1.1	3.5	4.5	1.4	–	2.5
iso-C_16∶0_ 3OH	2	1.0	2.1	1.6	1.9	tr	2.1	1.5
iso-C_17∶0_ 3OH	5	8.2	7.3	5.9	10.3	5.1	7.0	
**Branched**								
C_14∶0_ aldehyde	1.0	–	–	–	–	–	–	tr
iso-C_15∶0_	19	26.1	14.6	28.2	23.5	28.0	24.8	25.5
anteiso-C_15∶0_	1.0	1.3	3.0	3.2	tr	4.3	2.5	1.9
iso-C_15∶0_ aldehyde	2.0	3.2	1.2	1.3	tr	1.3	2.3	2.0
iso-C_15∶1_ G	6.0	2.9	7.4	3.7	5.8	7.2	5.0	5.0
iso-C_16∶0_	1	–	1.1	1.0	tr	1.0	–	1.1
iso-C_16∶1_ H	tr	–	1.0	1.0	tr	–	–	tr
iso-C_17∶1_ *ω*9c	3	6.0	5.2	4.3	4.1	6.0	1.1	2.9
**Unsaturated**								
C_15∶1_ *ω*6c	9	12.3	10.1	4.1	2.9	5.5	12.2	7.6
C_16∶1_ *ω*7c	10	3.7	11.2	19.2	15.7	18.1	2.2	9.8
C_17∶1_ *ω*6c	6	5.9	6.4	3.5	2.5	3.2	6.2	2.4
C_17∶1_ *ω*8c	1	1.0	1.5	–	tr	tr	1.3	tr
**Summed feature 1** a	–	–	–	2.0	1.7	1.4	–	1.7
**Unidentified fatty acid** b								
ECL 11.541	2	1.4	tr	tr	tr	tr	1.2	1.1
ECL 12.555	1	–	tr	–	–	–	1.1	tr
ECL 14.809	1	–	–	–	–	–	–	–
ECL 16.580	–	–	tr	–	1.1	–	–	tr

Taxa: 1, *F. plurextorum* 1126-1H-08^T^; 2, *F. pectinovorum* CCUG 58916^T^; 3, *F. aquidurense* CCUG 59847^T^; 4, *F. frigidimaris* CCUG 59364^T^; 5, *F. hydatis* DSM 2063^T^; 6, *F. araucananum* CCUG 61031^T^; 7, *F. chungangense* CCUG 58910^T^; 8, *F. oncorhynchi* CECT 7678^T^.

Values are percentages of total fatty acids; fatty acids representing less than 1% in all strains were omitted. tr = trace amount, i.e., <1%. - = not detected.

CFA values for type strains other than *F. plurextorum* 1126-1H-08^T^ were taken from the CCUG culture collection (http://www.ccug.se/). Strains were cultivated on the same medium and growth conditions.

a Summed features represent groups of two or three fatty acids that cannot be separated by GLC with the MIDI system. Summed feature 1 comprised iso-C_17∶1_ I/C_16∶0_ DMA.

b ECL, equivalent chain length.

The trout strains exhibited identical physiological and biochemical characteristics. Cells were Gram-negative rods, 0.7 µm wide and 3 µm long, non-endospore-forming, and non-gliding. Strains grew well under aerobic conditions and grew weakly under micro-aerobic conditions. Strains grew at 15–30°C with optimal growth at approximately 25°C, while no growth was observed at 37°C or 42°C. Growth occurred on trypticase-soy and nutrient agars but not on Marine agar after incubation at 25°C for 72 hours. Colonies were circular, yellow-pigmented, smooth and entire on TGE agar after 72 hours incubation at 25°C. Colonies are non-hemolytic on Columbia agar after 72 hours incubation at 25°C. Diffusible flexirubin-type pigments were produced and congo red was not absorbed by colonies. Growth did not occur in brain heart infusion broth containing 3, 4.5 and 6.5% NaCl. Catalase and oxidase were produced and nitrate and nitrite were reduced. Starch and tyrosine were degraded but DNA, gelatin, casein or agarose were not. A brown pigment was not produced on tyrosine agar. Aesculin was hydrolyzed but not urea, lecithin and arginine. Indole and H_2_S were not produced. Acid was not produced from D-glucose. Arabinose, D-glucose, mannose, N-acetyl-glucosamine, and maltose were used as sole carbon and energy sources but not citrate, mannitol, gluconate, caprate, adipate, and malate. Activities for alkaline phosphatase, leucine arylamidase, N-acetyl-β-glucosaminidase, *α*-glucosidase, acid phosphatase, and naphthol-AS-BI-phophohydrolase were detected. Esterase C4, valine arylamidase, *β*-galactosidase, ester lipase C8, lipase C14, cystine arylamidase, *α*-chymotrypsin, trypsin, *α*-galactosidase, *β*-glucuronidase, β-glucosidase, *α*-mannosidase and *α*-fucosidase were not detected.

The phenotypic characteristics that differentiated the trout strains from phylogenetically related species are shown in Table 2. The new species also can be also differentiated from the clinically relevant fish pathogens *F. columnare*, *F. psycrophilum* and *F. branchiophilum*, by the inability of these three species to grow in trypticase-soy agar and to hydrolyze aesculin [4]. Other species isolated from diseased fish such as *F. hydatis*, *F. jonshoniae* and *F. succinicans* are motile (gliding), degrade DNA and produce acid from carbohydrates [4], while the new species exhibited opposite results for those tests. Moreover, the new species can be readily differentiated from *F. chilense* and *F. araucananum* because the latter species are motile (gliding), grow in 3% NaCl and assimilate mannitol [9] and from *F. oncorhynchi* which produces β-galactosidase while the new species give opposite results for this test [10].

**Table 2:** Characteristics that differentiate *Flavobacterium plurextorum* sp. nov. from closely related *Flavobacterium* species based in the 16S rRNA tree topology.

Characteristic	1	2	3	4	5	6	7	8
Growth on Marine agar	**−**	**−**	**−**	**+**	**−**	**−**	**−**	**−**
Growth at 30°C	**+**	**+**	**+**	**−**	**+**	**+**	**+**	**+**
Hydrolysis of:								
L- tyrosine	**+**	**−**	**+**	**−**	**+**	**−**	**−**	**+**
DNA	**−**	**−**	**−**	**−**	**+**	**−**	**+**	**−**
Urea	**−**	**−**	**+**	**−**	**−**	**−**	**−**	**−**
Nitrate reduction	**+**	**+**	**−**	**−**	**+**	**+**	**+**	**+**
Assimilation of:								
Arabinose	**+**	**+**	**+**	**+**	**−**	**+**	**+**	**+**
Mannitol	**−**	**−**	**−**	**+**	**−**	**−**	**−**	**−**
N-acetyl-glucosamine	**+**	**+**	**+**	**+**	**+**	**+**	**-**	**+**
Production of:								
Valine arylamidase	**−**	**+**	**+**	**+**	**+**	**+**	**+**	**−**
* α*-Glucosidase	**+**	**−**	**−**	**+**	**+**	**+**	**−**	**+**
* β*-Glucosidase	**−**	**+**	**−**	**+**	**−**	**+**	**+**	**−**
N-Acetyl-*β*-glucosaminidase	**+**	**−**	**−**	**+**	**+**	**−**	**−**	**+**

Taxa: 1, *F. plurextorum* 1126-1H-08^T^; 2, *F. pectinovorum* CCUG 58916^T^; 3, *F. aquidurense* CCUG 59847^T^; 4, *F. frigidimaris* CCUG 59364^T^; 5, *F. hydatis* DSM 2063^T^; 6, *F. araucananum* CCUG 61031^T^; 7, *F. chungangense* CCUG 58910^T^; 8, *F. oncorhynchi* CECT 7678^T^.

Data are from this study.

+, positive reaction; −, negative reaction.

**Figure 2:**
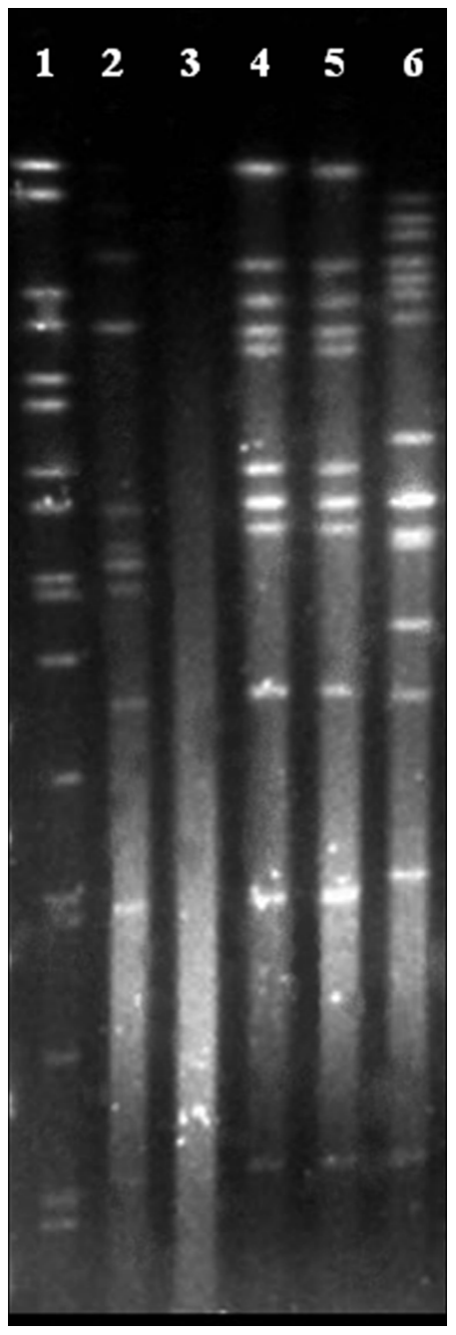
PFGE patterns generated after Bsp120I macrorestriction of *Flavobacterium plurextorum* sp. nov. Lane 1, DNA molecular size marker; Lanes 2 to 6, strains 1126-1H-08^T^, 51B-09, 986-08, 1084B-08 and 424-08, respectively.

After PFGE typing, the trout strains were characterized by 3 different restriction profiles with the enzymes *Bsp120*I (Fig. 2) and *Xho*I (not shown). Strains 986-08 and 1084B-08 exhibited indistinguishable restriction profiles with both enzymes and strain 51B-09 could not be characterized because its DNA systematically was autodegraded.

Flavobacteria are known to belong to the microbiota of fish and fish eggs [4], [5]. Therefore, although two strains were isolated from internal organs, the other three were recovered from gills and eggs which suggest that the new species could be saprophytic or commensal and able to colonize fish, and produce disease under stressful conditions or other predisposing circumstances such as coinfections with other bacteria or viruses, poor farming conditions or environmental disorders [4], [39]. This assumption should be confirmed by experimental infection trials. Nevertheless, the formal description of *Flavobacterium plurextorum* and the availability of tests to facilitate its identification from other *Flavobacterium* species associated with fish disease or isolated from diseased fish will aid laboratories in its recognition and identification in the future, and to improve the knowledge of its distribution and possible association with disease.

### Conclusion

The phylogenetic, genotypic and phenotypic results of the present polyphasic study demonstrated that the new strains isolated from rainbow trout represented a novel species of the genus *Flavobacterium*, for which the name *Flavobacterium plurextorum* sp. nov. is proposed (plu.rex.to’rum. L. comp. pl. plures, more, several, many; L. pl. n. exta -orum, entrails; N.L. gen. pl. n. plurextorum, of several internal organs). Detailed description of the morphological, physiological and biochemical characteristics of this species were indicated above. The type strain is 1126-1H-08^T^ ( = CECT 7844^T^ = CCUG 60112^T^).
