# Correction: *Flavobacterium plurextorum* sp. nov. Isolated from Farmed Rainbow Trout (*Oncorhynchus mykiss*)

**DOI:** 10.1371/annotation/6ab9387b-ebd3-4692-99e8-96661687b6bb

**Published:** 2013-07-31

**Authors:** Leydis Zamora, José F. Fernández-Garayzábal, Cristina Sánchez-Porro, Mari Angel Palacios, Edward R. B. Moore, Lucas Domínguez, Antonio Ventosa, Ana I. Vela

Results of the phenotypic, chemotaxonomic, genotypic and phylogenetic studies reveal that the new strains from rainbow trout characterized in this article constitute a novel species in the genus *Flavobacterium*, for which the name *Flavobacterium plurextorum* sp. nov. is proposed.

**Description of *Flavobacterium plurextorum* sp. nov.**

*Flavobacterium plurextorum* (plur. ex.to'rum. L. comp. pl. *plures*, more, several, many; L. pl. n. *exta -orum*, entrails; N.L. gen. pl. n. *plurextorum*, of several internal organs).

Cells are Gram-staining-negative rods, that measure approximately 0.7 µm wide and 3 µm long, and are non-endospore-forming, and non-gliding. Grows well under aerobic conditions and weakly under micro-aerobic conditions. Grows at 15-30ºC with optimal growth at approximately 25ºC, while no growth occurs neither at 37ºC nor at 42ºC. Growth occurs on trypticase-soy and nutrient agars but not on Marine agar after incubation at 25ºC for 72 hours. Growth does not occur in brain heart infusion broth containing 3.0, 4.5 and 6.5% (w/v) added NaCl. Colonies are circular, yellow-pigmented, smooth and entire on TGE agar after 72 hours incubation at 25ºC. Colonies are non-haemolytic on Columbia agar after 72 hours incubation at 25ºC. Diffusible flexirubin-type pigments are produced. Congo red is not absorbed by colonies. Positive for catalase and oxidase activities. Nitrate and nitrite are reduced. Starch and tyrosine are degraded but DNA, gelatin, casein or agarose are not. A brown pigment is not produced on tyrosine agar. Aesculin is hydrolysed but not urea, lecithin and arginine. Indole and H2S are not produced. Acid is not produced from D-glucose. Utilizes glucose, arabinose, mannose, N-acetyl-glucosamine, and maltose as sole carbon and energy sources but not citrate, mannitol, gluconate, caprate, adipate, and malate. Positive for alkaline phosphatase, leucine arylamidase, N-acetyl-β-glucosaminidase, α-glucosidase, acid phosphatase, and naphthol-AS-BI-phophohydrolase but negative for esterase C4, valine arylamidase, β-galactosidase, ester lipase C8, lipase C14, cystine arylamidase, α-chymotrypsin, trypsin, α-galactosidase, β-glucuronidase, β-glucosidase, α-mannosidase and α-fucosidase. The major cellular fatty acids of strain 1126-1H-08T are iso-C15:0 and C15:0. Cells contain menaquinone-6 (MK-6) as the major respiratory quinone.

The type strain, 1126-1H-08^T^ (=CECT 7844^T^ = CCUG 60112^T^), was isolated from an egg of a rainbow trout. The DNA G+C content of this strain is 33.2 mol%.

